# Depth-Based Detection of Standing-Pigs in Moving Noise Environments

**DOI:** 10.3390/s17122757

**Published:** 2017-11-29

**Authors:** Jinseong Kim, Yeonwoo Chung, Younchang Choi, Jaewon Sa, Heegon Kim, Yongwha Chung, Daihee Park, Hakjae Kim

**Affiliations:** 1 Department of Computer and Information Science, Korea University, Sejong City 30019, Korea; skykeeop@korea.ac.kr (J.K.); ycc4477@korea.ac.kr (Y.C.); sjwon92@korea.ac.kr (J.S.); khg86@korea.ac.kr (H.K.); dhpark@korea.ac.kr (D.P.); 2 Department of Applied Statistics, Korea University, Sejong City 30019, Korea; william0516@korea.ac.kr; 3 Class Act Co., Ltd., Digital-ro, Geumcheon-gu, Seoul 08589, Korea; krunivs@gmail.com

**Keywords:** agriculture IT, computer vision, foreground detection, depth information, moving noise

## Abstract

In a surveillance camera environment, the detection of standing-pigs in real-time is an important issue towards the final goal of 24-h tracking of individual pigs. In this study, we focus on depth-based detection of standing-pigs with “moving noises”, which appear every night in a commercial pig farm, but have not been reported yet. We first apply a spatiotemporal interpolation technique to remove the moving noises occurring in the depth images. Then, we detect the standing-pigs by utilizing the undefined depth values around them. Our experimental results show that this method is effective for detecting standing-pigs at night, in terms of both cost-effectiveness (using a low-cost Kinect depth sensor) and accuracy (i.e., 94.47%), even with severe moving noises occluding up to half of an input depth image. Furthermore, without any time-consuming technique, the proposed method can be executed in real-time.

## 1. Introduction

The early detection of management problems related to health and welfare is an important aspect of caring for group-housed livestock. In particular, caring for individual animals is necessary to minimize the possible damage caused by infectious diseases or other health and welfare problems. However, it is almost impossible for individual animals to be cared for by a small number of farm workers who work on a large-scale livestock farm. For example, the pig farm from which we obtained video monitoring data in Korea had more than 2000 pigs per farm worker. 

Several studies using surveillance techniques have recently been conducted to automatically monitor livestock, in what is known as “precision livestock farming” (PLF) [1]. Several attached sensors, such as accelerometers, gyro sensors, and radio frequency identification (RFID) tags, are used to automate the management of livestock farms in examples of PLF [2]. However, such approaches increase costs, and require additional manual labor for activities such as the attachment and detachment of sensors to and from individual animals by farm administrators. To circumvent this, studies have been conducted that analyze data from non-attached (i.e., non-invasive) sensors (such as cameras) [2,3,4,5]. In this study, we focus only on video-based pig monitoring applications [6].

In fact, video-based pig monitoring applications have been reported since 1990 [7,8]. However, because of the practical difficulties (e.g., light fluctuation, shadowing, cluttered background, varying floor status caused by urine/manure, etc.) presented by commercial farms, even the accurate detection of pigs in commercial environments has remained a challenging problem until now [9,10,11,12,13,14,15,16,17,18,19,20,21,22,23,24,25,26,27,28,29,30,31,32,33,34,35,36,37,38,39,40,41,42,43]. To consider these practical difficulties, it is reasonable to employ a topview-based depth sensor. However, the depth values obtained from a low-cost sensor such as Microsoft Kinect may be inaccurate for classifying a weaning pig as standing or lying. Furthermore, in many monitoring applications, the input video stream data needs to be processed in real-time for an online analysis. 

In this study, we propose a low-cost, practical, and real-time method for detecting standing-pigs at night, with the final goal of achieving 24 h individual pig tracking in a commercial pig farm. In particular, caring for weaning pigs (25 days old) is the most important issue in pig management, because of their weak immunity. Therefore, we aim to develop a method for detecting standing-pigs in a pig pen during a one month period after weaning (i.e., 25 days–55 days old). Compared with previous work, the contributions of the proposed method can be summarized as follows:

- Standing-pigs are detected at night (i.e., with a light turned off) with a low-cost depth camera. It is well known that most pigs sleep at night [44,45,46]. For the purpose of 24 h individual pig tracking, we only need to detect standing-pigs (i.e., we do not need to detect the majority of lying-pigs at night). Recently, low-cost depth cameras, such as Microsoft Kinect, have been released, and thus we can detect standing-pigs using depth information. However, the size of a 20-kg weaning pig is much smaller than that of a 100-kg adult pig. Furthermore, the accuracy of the depth data measured from a topview Kinect degrades significantly, because there is a limited distance (e.g., a maximum range of 4.5 m) and field-of-view (e.g., horizontal degree of 70.6 and vertical degree of 60) in which depth values are covered. If we install a Kinect at 3.8 m above the floor to cover the entire area of a pen (i.e., 2.4 m × 2.7 m), thus minimizing the installation cost for a large-scale farm, then it is difficult to classify a weaning pig as standing or lying. To increase the accuracy, we consider the undefined depth values around standing-pigs.
- A practical issue caused by moving noises is resolved. For example, in a commercial pig farm with a harsh environment (i.e., disturbances from dust and dirt), there are many moving noises (i.e., undefined depth values varying across frames) at night. Because these moving noises occlude pigs (i.e., even up to half of a scene can be occluded by moving noises), we need to recover the depth values that are occluded by the moving noises. Because we utilize the undefined depth values around standing-pigs to increase the detection accuracy, we need to classify undefined depth values as useful ones (i.e., caused by standing-pigs) and useless ones (i.e., caused by moving noises). We apply spatial and temporal interpolation techniques to reduce the moving noises. In addition, we combine the detection results of standing-pigs from the interpolated images and the undefined depth values around standing-pigs to detect standing-pigs more accurately.
- A real-time solution is proposed. Detecting standing-pigs is a basic low-level vision task for intermediate-level vision tasks such as tracking and/or high-level vision tasks such as aggressive analysis. To complete the entire vision tasks in real-time, we need to decrease the computational workload of the detection task. Without any time-consuming techniques to improve the accuracy of depth values, we can detect standing-pigs accurately at a processing speed of 494 frames per second (fps).

The remainder of this paper is structured as follows. Section 2 summarizes topview-based pig monitoring results, targeted for commercial farms. Section 3 describes the proposed method for detecting standing-pigs in various noise environments, including with moving noises. The experimental results are presented in Section 4, and conclusions are presented in Section 5.

## 2. Background

As explained in Section 1, the accurate detection of pigs in commercial environments has been a challenging problem since 1990, because of the practical difficulties (e.g., light fluctuation, shadowing, cluttered background, varying floor status caused by urine/manure, etc.) presented by commercial farms. Table 1 summarizes the topview-based pig monitoring results introduced recently [9,10,11,12,13,14,15,16,17,18,19,20,21,22,23,24,25,26,27,28,29,30,31,32,33,34,35,36,37,38,39,40,41,42,43]. Two-dimensional gray-scale or color information has been used to detect a single pig in a pen or a specially built facility (i.e., in “constrained” environments) [9,10,11]. However, even with advanced techniques applied to 2D gray-scale or color information, it remains challenging to detect multiple pigs accurately in a “commercial” farm environment [12,13,14,15,16,17,18,19,20,21,22,23,24,25,26,27,28,29,30,31,32,33]. For example, images from a gray-scale or RGB camera are affected by various illuminations in a pig pen. Thus, a monitoring system based on a gray-scale or RGB camera cannot detect objects in low- to no-light conditions. Although some monitoring results at night have been reported using infrared cameras [34,35,36], problems caused by a cluttered background cannot be perfectly solved. Although some researchers have utilized a thermal camera to resolve the cluttered background problem [37], this is an expensive solution for large-scale farms. 

**Figure 1:**
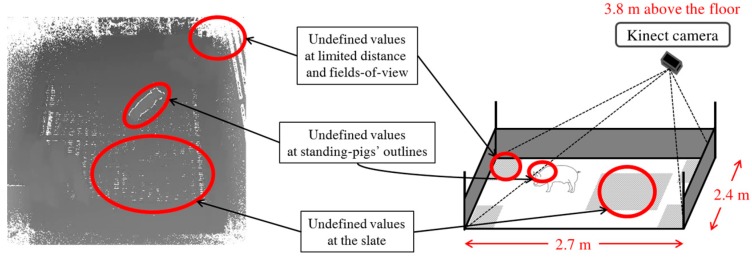
Undefined values caused by various factors in the monitoring environment in a commercial farm.

To solve the cluttered background problem for 2D information, some researchers have utilized a stereo camera [38]. However, the accuracy measured from a stereo camera is far from a level at which 24 h individual pig tracking is possible, even with many pigs in a pen. Recently, low-cost depth cameras such as Kinect have been released. Compared with typical stereo-camera-based solutions, a Kinect can provide more accurate depth information at a much lower cost, without a heavy computational workload [39,40,41,42,43]. In principle, Kinect cameras can recognize whether pigs are lying or standing based on the depth data measured. However, a low-cost Kinect camera has a limited distance range (i.e., up to 4.5 m), and the accuracy of the depth data measured by a Kinect decreases quadratically as the distance increases [47]. Thus, the accuracy of the depth data measured by a Kinect degrades significantly when the distance between it and a pig is larger than 3.8 m. Furthermore, the slate-based floor of a pig pen generates many undefined depth values, because of the field-of-view of the installed Kinect. A further issue is that a greater number of undefined depth values appear at the top of a depth image (see Figure 1). Because of the ceiling structure of the pig pen in a commercial farm in which we installed a Kinect, the Kinect could not be installed at the center of the pig pen. Considering these difficulties, it is challenging to classify a 20-kg weaning pig as standing or lying using a Kinect camera installed 3.8 m above the floor. Figure 1 shows the limitations caused by the characteristics of the Kinect camera and the pig pen.

**Figure 2:**
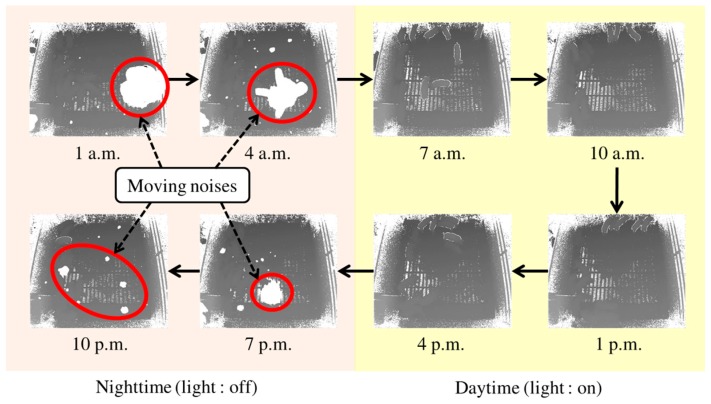
Daytime and nighttime images obtained from a 3D depth camera. Moving noises, shown as large white regions, can be observed at night.

In this study, we consider moving noises at night (see Figure 2) further. In a commercial farm, we could observe many moving noises every night, and even up to half of a scene was occluded by moving noises. For 24 h individual pig tracking in a commercial pig farm, we need to resolve this type of practical problem. To the best of our knowledge, this is the first report on handling these types of moving noises obtained from a commercial pig farm at night through a Kinect.

A final comment regarding previous research concerns real-time monitoring. Although online monitoring applications should satisfy the real-time requirement, many previous results did not specify the processing speed, or could not satisfy the real-time requirement (see Table 1). By carefully balancing the tradeoff between the computational workload and accuracy, we propose a light-weight detection method with an acceptable accuracy for the final goal of achieving a real-time “complete” vision system, consisting of intermediate- and high-level vision tasks, in addition to low-level vision tasks. 

## 3. Proposed Approach

We initially define the terms used in the proposed method, to enhance the readability. Table 2 explains the main terms for each process. 

**Figure 3:**
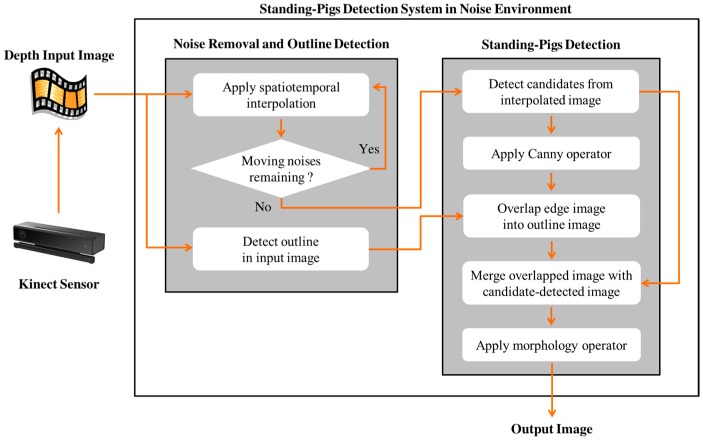
Overview of the proposed method.

To detect standing-pigs at night in a pig pen, it is desirable to utilize a depth sensor, such as a Kinect camera. This allows the sensor to gain depth information on pigs (i.e., the distance from a pig to the camera) without light influences, such as the light being turned on or off in a pig pen. However, because much dirt or dust may be generated at night in the pen, many moving noises appear in a video stream obtained from the depth sensor. These noises make it difficult to detect standing-pigs due to occlusions on them. Therefore, we propose a method to effectively remove the noises generated from dirt or dust in the video, and to precisely detect standing-pigs using undefined depth values (e.g., outlines) of standing-pigs. Figure 3 presents the overview of our detection method for standing-pigs at night. 

### 3.1. Noise Removal and Outline Detection

Using depth values from a 3D Kinect camera, information on pigs can be obtained at night without a light in a pen. However, undefined depth values corresponding to moving noises (i.e., $UDF_{moving}$) emerged in this process due to the dirt or dust generated from pigs, and this disturbs the accurate detection of pigs. To remove these noises, an interpolation technique using spatiotemporal information is applied to the input video. 

**Figure 4:**
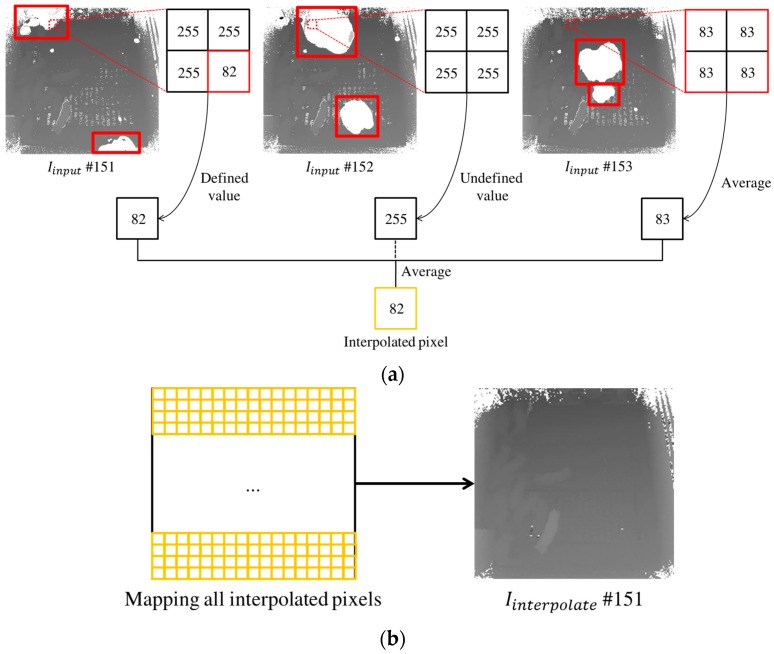
Applying the interpolation technique to remove undefined values in three consecutive images: (**a**) an interpolated pixel is produced by averaging over consecutive images except for undefined values, where moving noises are represented as bold boxes; and (**b**) $I_{interpolate}$ is produced by integrating all interpolated pixels.

Initially, an interpolation technique using a 2 × 2 window is applied to a current image, with two consecutive images (i.e., using temporal information), in $I_{input}$. As shown in Figure 4a, the 2 × 2 window is used as spatial information. The 2 × 2 window moves within $I_{input}$, and performs the interpolation on every pixel in $I_{input}$. The interpolation is performed in three cases according to the pixel attributes in the window. In the first case, if more than two pixels in the 2 × 2 window have defined depth values such as right of Figure 4a, then an interpolated pixel can be created through their average calculation. In the second case, if there is only one pixel as a defined depth value in the window such as left of Figure 4a, then the pixel can be specified as an interpolated pixel. In the third case, if all pixels in the window are undefined such as middle of Figure 4a, then an interpolated pixel is assigned as an undefined depth value (i.e., noise pixel). In this procedure, three interpolated pixels obtained from each image are merged as a definitive interpolated pixel by calculating an average over them. Note that an undefined depth value is not included in the average calculation. Here, $I_{interpolate}$ is produced by integrating all of the interpolated pixels derived from all pixels in the input image. That is, $UDF_{moving}$ can be removed by repeating the interpolation technique for all of the images in $I_{input}$. 

**Figure 5:**
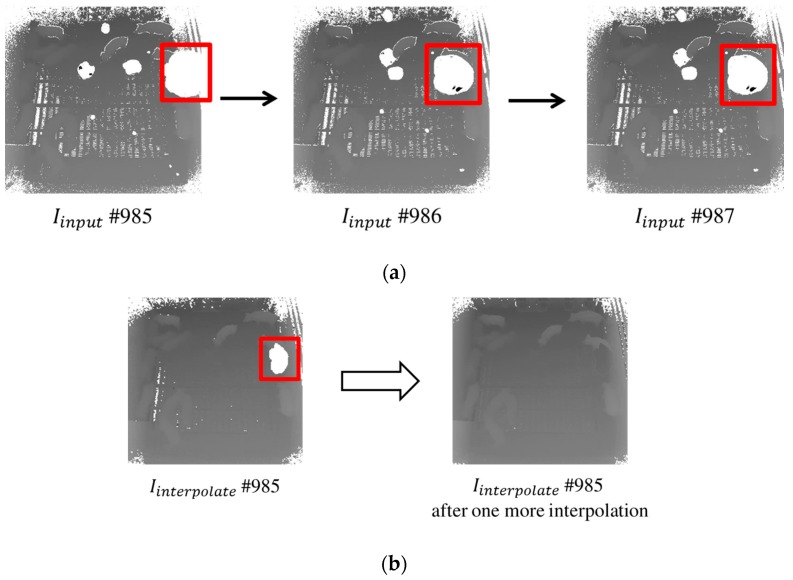
Problem in which noises are not removed with one interpolation and its solution: (**a**) relatively slow $UDF_{moving}$ in consecutive images; and (**b**) resulting image from applying the interpolation technique one more time.

Although most $UDF_{moving}$ areas usually move fast (see the bold boxes in Figure 4a), there are relatively slow moving $UDF_{moving}$ areas in certain consecutive images. In contrast with Figure 4b, some of these relatively slow $UDF_{moving}$ areas are not entirely removed by applying one spatiotemporal interpolation (see Figure 5b). This problem is due to the duplication of coordinates of the noises in consecutive images, and thus the interpolated pixels at such coordinates are continuously calculated as an undefined value. 

To resolve this problem, the remaining noises in $I_{interpolate}$ can be removed by applying the interpolation one more time. A pixel in the preceding image is checked at the same coordinate corresponding to $I_{interpolate}$, and it is mapped into $I_{interpolate}$ if it is recognized as a defined depth value. However, if the pixel has an undefined depth value, this procedure is repeated until the value at that coordinate is not an undefined depth value. Figure 5 illustrates the problem and its solution for relatively slow moving noises, which are entirely removed by applying the spatiotemporal interpolation technique one more time.

**Figure 6:**
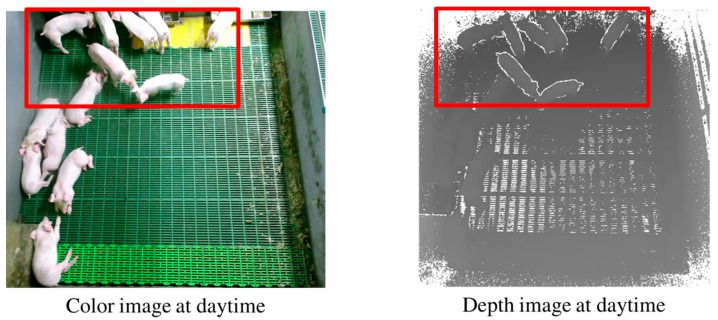
Standing-pigs within the bold box have undefined outlines.

Furthermore, depth values are not consistent for all pigs, owing to different growth rates. For example, even if all of the pigs in a pig pen are weaning pigs (25 days old), a well-grown pig may often be larger than the others. In the depth image, the larger weaning pig may appear to be a standing-pig when it is actually sitting on the floor. To resolve this difficulty, we exploit $UDF_{outline}$ generated around standing-pigs. Because the distance between a weaning lying-pig and the floor is small, $UDF_{outline}$ values are not observed around a lying-pig. However, even for weaning pigs, $UDF_{outline}$ values are observed around standing-pigs. Figure 6 shows that standing-pigs have $UDF_{outline}$ values, but lying-pigs do not. Note that Figure 6 displays both color and depth images at daytime, to verify that the undefined outlines are generated around standing-pigs only. 

**Figure 7:**
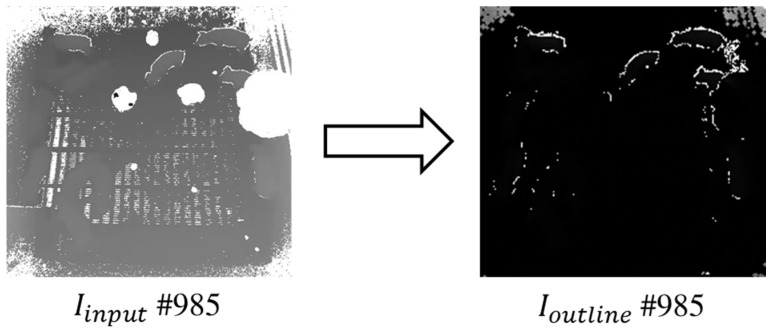
Result of detecting $UDF_{outline}$ around standing-pigs.

Therefore, $UDF_{outline}$ can be used as beneficial information to detect standing-pigs, even though $UDF_{outline}$ occurs due to the limitation of the Kinect camera in $I_{input}$. However, because $UDF_{outline}$ areas have the same values as other undefined values (i.e., 255), these are also removed after the interpolation technique. Thus, it is necessary to distinguish between $UDF_{outline}$ and other undefined values. To distinguish $UDF_{outline}$, we exploit the differences between widths of $UDF_{outline}$ and other undefined values. For example, most areas with undefined values have widths that are greater than three, whereas $UDF_{outline}$ area has widths of less than two. These attributes help to accurately distinguish $UDF_{outline}$ from the others. First, 3 × 3 neighboring pixel values are compared to confirm whether they are $UDF_{outline}$ or not. Then, if the total pixels contain fewer than two undefined values, they are regarded as $UDF_{outline}$. Figure 7 shows that fewer than two undefined values in $I_{input}$ are detected as $UDF_{outline}$.

### 3.2. Detection of Standing-Pigs 

**Figure 8:**
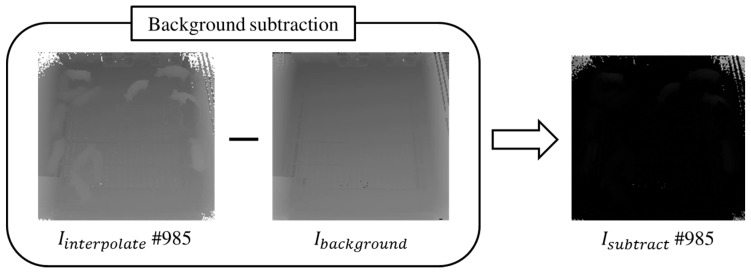
Result of background subtraction.

After removing $UDF_{moving}$ using the spatiotemporal interpolation technique, the depth values in $I_{interpolate}$ are subtracted from $I_{background}$. Because the distance from each pig to the camera is different depending on the location of the pig, the depth values of pigs obtained from the Kinect camera need to be subtracted from $I_{background}$. Ideally, the depth values obtained from a location under the same condition should be consistent; however, the depth values obtained by a low-cost Kinect are not consistent. For example, for the same location, different depth values of 76, 112 and 96 are obtained as time progresses. To solve this inconsistency problem, $I_{background}$ can be generated carefully as follows. Initially, a depth video in the empty pen is acquired for ten minutes. Then, the spatial interpolation is applied to $I_{input}$ to remove undefined values such as $UDF_{floor}$ and $UDF_{limitation}$. Furthermore, we compute the most frequent depth values of each pixel in $I_{input}$ over ten minutes. However, for certain pixel locations within a floor, the resulting values may not be similar to those of adjacent pixels. To resolve this problem, we apply line-filling, which replaces such a value with the average of the adjacent values in the same row, in order to obtain $I_{background}$. Figure 8 shows the result of the background subtraction for depth values in $I_{interpolate}$.

**Figure 9:**
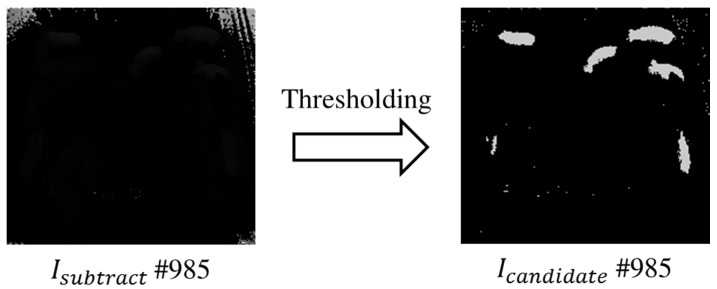
Candidates detected as standing-pigs.

From $I_{subtract}$, candidates for standing-pigs are detected by using a thresholding technique for depth values. By analyzing $I_{subtract}$ images, we found that the depth values for standing- and lying-pigs have some overlapping ranges. If the depth values do not overlap, then we can simply set a threshold to distinguish between standing- and lying-pigs. However, to resolve the overlapping problem, we generate standing pig candidates $I_{candidate}$, and then verify these with the edge information $I_{edge}$ from the candidates and the outline information $UDF_{outline}$ for standing-pigs. First, we can obtain $I_{candidate}$ by detecting candidates in $I_{subtract}$ that may be considered as standing-pigs by setting a threshold. In addition, by using the thresholding technique, some undefined values resulting from limitations of the monitoring environment can be removed. That is, the undefined values such as $UDF_{floor}$ and $UDF_{limitation}$ are removed through the thresholding technique. Figure 9 shows candidates detected as standing pigs, as well as unnecessary undefined values removed through the thresholding in $I_{subtract}$. 

**Figure 10:**
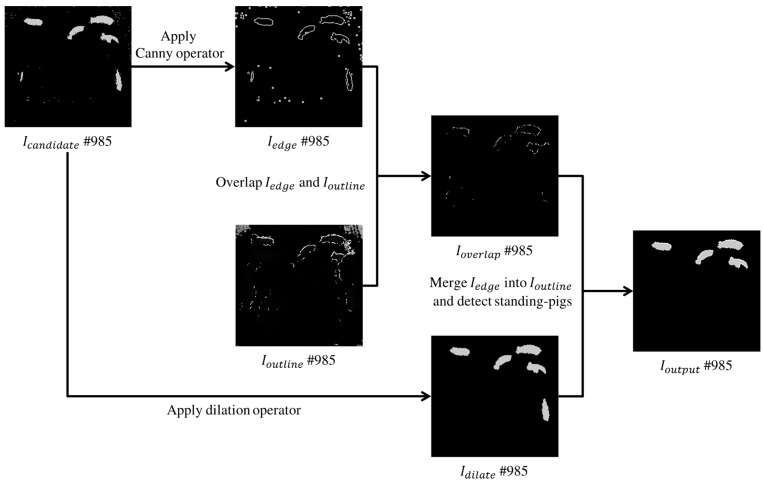
Total procedure for detecting standing-pigs with an example image #985.

**Figure 11:**
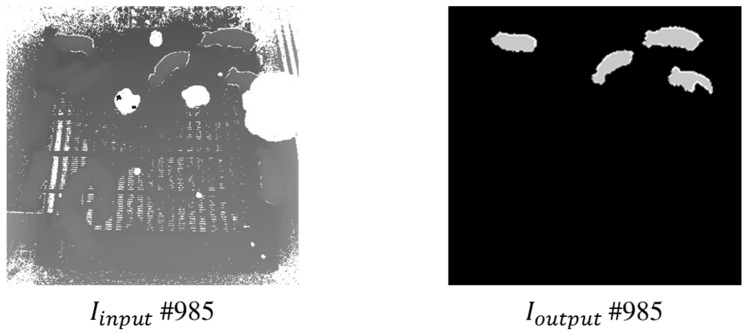
Results of standing-pigs detection from $I_{input}$ to $I_{output}$.

Based on both $I_{candidate}$ and $I_{outline}$, if $UDF_{outline}$ is applied to $I_{candidate}$, then standing-pigs in the pig pen can be identified more accurately. First, the candidates’ edges (i.e., $I_{edge}$) can be derived using a Canny operator. In fact, $I_{outline}$ explained in Section 3.1 includes not only $UDF_{outline}$, but also other undefined values. To derive a more accurate set of $UDF_{outline}$, the candidates’ edges in $I_{edge}$ are overlapped into $I_{outline}$. Then, a dilation operator is applied to the candidates in $I_{candidate}$, to eventually detect them as standing-pigs using the more accurate $UDF_{outline}$ in $I_{outline}$. Finally, the more accurate $UDF_{outline}$ values in $I_{overlap}$ are combined with $I_{dilate}$. In $I_{merge}$, standing-pigs can be detected by calculating an overlapping ratio between the dilated candidates and the more accurate $UDF_{outline}$. In other words, if the boundaries of a dilated candidate overlap with the pixels of the more accurate $UDF_{outline}$ by more than 50%, then the candidate can be identified as a standing-pig in $I_{output}$. Figure 10 summarizes the procedures for detecting standing-pigs using both $UDF_{outline}$ in $I_{outline}$ and candidates in $I_{candidate}$, and Figure 11 shows the detection result for standing-pigs in the pig pen.

Finally, the proposed method is summarized in Algorithm 1, given below.

**Algorithm 1** Standing-pigs detection algorithmInput: Depth Image Output: Detected Image Step 1: While moving noise remainingApply spatiotemporal interpolation;Subtract $I_{background}$ with $I_{interpolate}$;
Step 2:
If widths of undefined values ≤ 2: Determine as an outline;Else:Determine as a noise and remove it on the area;
Step 3:
If *threshold1* ≤ subtracted pixel value ≤ *threshold2*: Determine as candidates for standing-pigs;Else: Determine as a noise and remove it on the area;Detect edges of candidates;
Step 4:
Overlap $I_{edge}$ into $I_{outline}$;If outline and edge on the same area:  Determine as an outline;Else: Determine as a noise and remove it on the area;
Step 5:
Merge $I_{overlap}$ with $I_{candidate}$;If candidate pigs touch outlines:Detect standing-pigs;Else:Determine as a noise and remove it on the area;

## 4. Experimental Results

### 4.1. Experimental Environments and Dataset 

In our experiment, the proposed method was evaluated using Intel Core i7-7700K 4.20 GHz (Intel, Santa Clara, CA, USA), 32 GB RAM, Ubuntu 16.04.2 LTS (Canonical Ltd, London, UK), and OpenCV 3.2 [48] for image processing. We installed a topview Kinect camera (Version 2.0, Microsoft, Redmond, WA, USA) on a ceiling at a height of 3.8 m in a 2.4 m × 2.7 m pig pen located in Sejong Metropolitan City, Korea. 

In the pig pen, we simultaneously obtained color and depth videos from 13 weaning pigs (i.e., 25 days old) through the Kinect camera. The color video had a resolution of 960 × 540 and 30 frames per second (fps), while the depth video had a resolution of 512 × 424 and 30 fps. 

As described in Section 3, it was impossible to detect standing-pigs in the color video, because a light in the pig pen was turned off at night. Therefore, we only exploited the depth video, which could be used to monitor pigs at night. We used 8 h of depth video, including daytime (07:00, 10:00, 13:00 and 16:00) and nighttime (01:00, 04:00, 19:00 and 22:00), which consisted of 480 depth images (one image per minute). Because it was highly time consuming to create ground truth data, especially for nighttime images (i.e., when the light was turned off), we selected one image for each minute as a representative image. We then applied the proposed method to all the images to detect standing-pigs in the pen.

### 4.2. Detection of Standing-Pigs under Moving Noise Environment 

Before detecting standing-pigs in the pig pen, we removed moving noises using the spatiotemporal interpolation technique. As explained in Section 3.1, we sequentially exploited spatial information to remove the moving noises. Moreover, we used temporal information to remove certain problematic noises, such as relatively slow moving noises. Then, 480 $I_{interpolate}$ images were obtained by applying the interpolation technique to 1440 $I_{input}$ images. From $I_{interpolate}$, we obtained 480 $I_{subtract}$ images by using background subtraction with $I_{background}$, and then obtained $I_{candidate}$ to detect candidates by applying the thresholding technique to $I_{subtract}$. 

**Figure 12:**
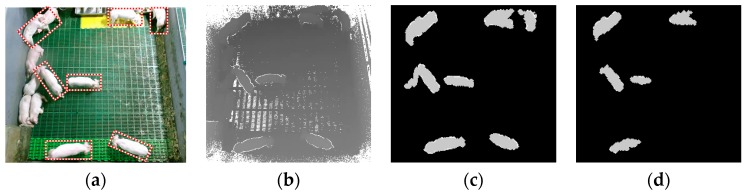
Differences of detecting standing-pigs according to *threshold1*: (**a**) color image; (**b**) depth image; (**c**) detection of standing-pigs with *threshold1* = 9; and (**d**) detection of standing-pigs with *threshold1* = 15.

For detecting the candidates, the defined depth values for standing- and lying-pigs in $I_{subtract}$ were measured as 9–30 and 4–15, respectively. In fact, the range of depth values for standing- and lying-pigs overlapped, and a lying-pig in the overlapping interval might be detected as a standing-pig. However, because our final goal is to implement a 24 h tracking system for pigs in the pen, it is not a serious problem to detect some lying-pigs as standing-pigs. Thus, we set *threshold1* to 9, to detect all the standing-pigs without missing any. In addition, we set *threshold2* to 30 to remove the remaining undefined values. That is, if the depth values were greater than *threshold1*, then the depth values were detected as candidates for standing-pigs. Moreover, if the depth values were greater than *threshold2*, then the remaining undefined values were removed. Figure 12 shows differences of detecting standing-pigs according to *threshold1*. As shown in Figure 12c,d, all the standing-pigs could be detected by setting *threshold1* to 9.

**Figure 13:**
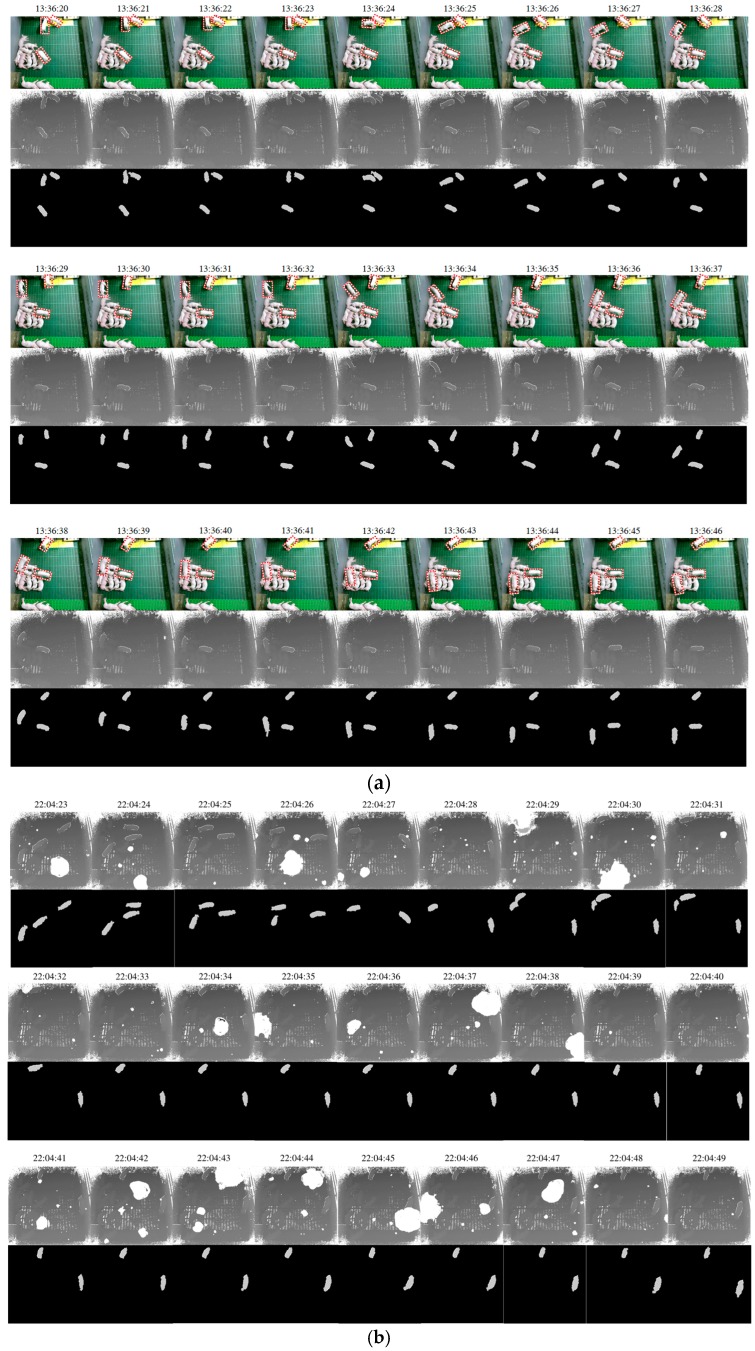
Results of detection of standing-pigs during the daytime and nighttime: (**a**) detected standing-pigs during daytime (13:36:20–13:36:46); and (**b**) detected standing-pigs during nighttime (22:04:23–22:04:49). Because a light was turned off, corresponding color images are not shown during nighttime.

To identify the standing-pigs among detected candidates, $UDF_{outline}$ in $I_{input}$ was overlapped with edges of the candidates. This was conducted to identify the more accurate $UDF_{outline}$ of a standing-pig if the edges in a region of a candidate matched $UDF_{outline}$ in $I_{input}$. If the candidates overlapped with the actual $UDF_{outline}$, then we finally identified the standing-pigs in these regions. Figure 13 displays the results for the detection of standing-pigs during the daytime and nighttime. 

### 4.3. Evaluation of Detection Performance

To evaluate the detection performance of the proposed method, we compared the number of standing-pigs detected using our method with that of existing methods for object detection, which included the Otsu algorithm [49] (i.e., well-known method for object detection) and YOLO9000 [50] (i.e., a recently-used method for object detection based on deep learning). 

In case of the Otsu algorithm, a background image was created by using the average and minimum values of each pixel in the input images for ten minutes from the empty pig pen. Using the test images, background subtraction was applied, and then the Otsu algorithm was performed. It is well known that the background subtraction method using the minimum value can detect typical foregrounds accurately with a Kinect camera [51]. However, as explained in Section 2 and Section 3, there are many difficulties in detecting standing-pigs after weaning. That is, we confirmed that standing-pigs in the pen could not be detected at all, because the Otsu algorithm binarized results into undefined and defined regions such as pigs, floor, and side-walls. 

**Figure 14:**
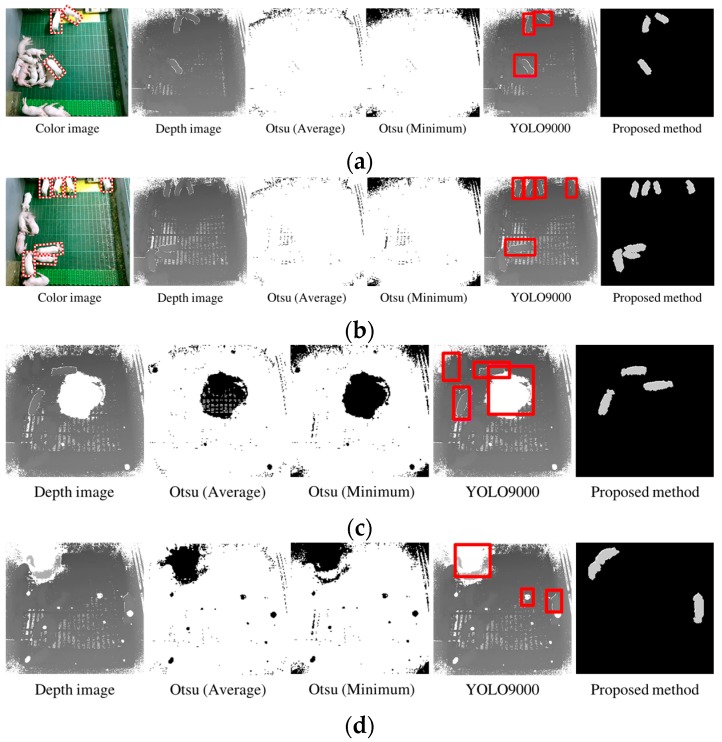
Results of each method for detecting standing-pigs: (**a**,**b**) results during daytime; and (**c**,**d**) results during nighttime. Because the light was turned off, corresponding color images are not shown during nighttime.

In the case of YOLO9000, we generated a model using the training data, which consisted of 600 depth images. We set some parameters of YOLO9000 as follows: 0.001 for learning rate, 0.9 for momentum, 0.0005 for decay, leaky ReLU as the activation function, and 10,000 for the epoch. From each test image, YOLO9000 produced bounding boxes to represent standing-pigs, and the confidence score was calculated to measure the similarity between the training model and the bounding boxes produced from YOLO9000. This score was used to detect the target objects (i.e., standing-pigs) among the bounding boxes, by using a threshold in YOLO9000. We exploited the default threshold of 0.24 to detect standing-pigs in YOLO9000. It is well known that YOLO9000 can detect typical foregrounds accurately in real-time [52]. However, YOLO9000 produced many false-positive and false-negative bounding boxes in detecting standing-pigs. Figure 14 displays the results of the standing-pigs detection for each method. 

As shown in Figure 14, the Otsu method could not detect standing-pigs at all, and thus we did not compute the accuracy of the Otsu method. In fact, the Otsu algorithm has been performed using a histogram distribution to classify as the background, and with the objects in an input image. However, in our case, the depth values between the background and the objects were similar, and the depth values of the noises had some differences with the objects. In addition, because the Otsu algorithm binarized the background and objects as the same group, the pigs could not be detected using the Otsu algorithm. Meanwhile, YOLO9000 is a recent method for object detection. As YOLO9000 imitates the process in which the human brain receives visual information, it learns the feature vectors optimized for training samples by themselves, and improves the performance of object classification by using these. Therefore, we compared the detection accuracy of the proposed method with that of YOLO9000.

In the experimental results for the proposed method and YOLO9000, we calculated the detection accuracy for standing-pigs to compare the performance of each method. The detection accuracy was calculated for each method using the equation below:

(1) $$\mathrm{Accuracy}=\left(1-\frac{\mathrm{FP}+\mathrm{FN}}{\mathrm{TP}+\mathrm{FN}}\right)\times 100$$

where true positive (TP) is “standing-pigs” identified as “standing-pigs”, true negative (TN) is “lying-pigs or noises” identified as “not standing-pigs”, false positive (FP) is “lying-pigs or noises” identified as “standing-pigs”, and false negative (FN) is “standing-pigs” identified as “lying-pigs or noises”, respectively. In particular, for each standing-pig, if the detected result had more than 50% intersection-over-union (IoU) [53] with the ground truth, then it was regarded as TP. Otherwise, it was regarded as FN. In Equation (1), the denominator (i.e., TP + FN) represents the number of standing-pigs, and the numerator (i.e., FP + FN) represents the number of detection failures. That is, the accuracy is comprised of how many pigs are failed to be detected as standing- or lying-pigs among the actual standing-pigs.

Based on the experimental results, the detection accuracies for standing-pigs were measured as 94.47% (proposed method) and 86.25% (YOLO9000 method) as shown in Table 3. In Table 4, the number of undefined pixels means the average percentage of undefined pixels from the total number of pixels of $I_{input}$. Even if this comprised more than 20% of the input image, it was possible to detect standing-pigs with a higher accuracy using the proposed method. Because we set *threshold1* to 9, we could detect all the standing-pigs using the proposed method. As shown in Figure 14c,d, we could even detect standing-pigs occluded by moving noises, by applying the spatiotemporal interpolation. Furthermore, all the false standing-pigs detected were lying-pigs (having distance values overlapped with standing-pigs). On the contrary, with YOLO9000, some of standing-pigs were missed, and thus 24-h individual pig tracking might not be possible with this method. In addition, the false standing-pigs detected by YOLO9000 consisted of the floor or moving noises as well as lying-pigs (see Figure 14).

Furthermore, we measured the execution time of each method, in order to confirm the real-time performance of standing-pig detection. As a result, the proposed method provided a faster processing speed in detecting standing-pigs than that of YOLO9000. Table 5 presents the processing speeds of each method for detecting standing-pigs. As explained in Section 1, our final goal is to develop a complete monitoring system, including both intermediate- and high-level vision tasks in real-time. By considering the further procedures in both intermediate- and high-level vision tasks, the detection of standing-pigs needs to be executed as fast as possible. Without time-consuming techniques (i.e., at least few seconds are required to process a single depth image to improve inaccurate depth values) such as in [54,55], it is possible to develop a real-time pig monitoring system including both intermediate- and high-level vision tasks.

## 5. Conclusions 

The automatic detection of standing-pigs in a surveillance camera environment is an important issue for the efficient management of pig farms. However, standing-pigs could not be detected accurately at night on a commercial pig farm, even using a depth camera, owing to moving noises. 

In this study, we focused on detecting standing-pigs in real-time in a moving noise environment to analyze individual pigs with the ultimate goal of 24-h continuous monitoring. That is, we proposed a method to detect standing-pigs at night without any time-consuming techniques. In the preprocessing step, the noise in the depth image was removed by applying a spatiotemporal interpolation technique, to alleviate the limitations of a low-cost depth camera such as Kinect. Then, we detected the standing-pigs by carefully generating a background image and then applying a background subtraction technique. In particular, we utilized undefined outline information (i.e., the undefined depth values around standing-pigs) to detect standing-pigs in a moving noise environment. 

Based on the experimental results for 480 video images (including 1186 standing-pigs) over eight hours (i.e., obtained during 01:00–10:00 and 13:00–22:00 in intervals of three hours), we could correctly detect all 1186 standing-pigs (while the ground truth-based accuracy was 94.47%) in real-time. As a future work, we will use the infrared information obtained from a Kinect sensor to improve the detection accuracy further. In addition, we will also consider the case of monitoring a large pig room by using multiple Kinect sensors. By extending this study, we will develop a real-time 24-h individual pig tracking system for the final goal of individual pig care. 

## Figures and Tables

**Table 1:** Topview-based pig monitoring results (published during 2011–2017) targeted for commercial farms.

Information	Camera Type	No. of Pigs in a Pen	Pig Type	Classification between Standing and Lying Postures	Management of Moving Noise	Processing Speed (fps)	Reference
2D	Color	1	Fattening Pig	No	No	Not Specified	[9]
	Gray-Scale	1	Sow	No	No	1.0	[10]
	Gray-Scale	1	Sow	No	No	2.0	[11]
	Gray-Scale	Not Specified	Sow + Piglets	No	No	4.0	[12]
	Color	9	Piglets	No	No	Not Specified	[13]
	Color	12	Piglets	No	No	4.5	[14]
	Color	11	Fattening Pigs	No	No	1.0	[15]
	Gray-Scale	2–12	Piglets	No	No	Not Specified	[16]
	Color	7	Not Specified	No	No	Not Specified	[17]
	Color	7	Not Specified	No	No	Not Specified	[18]
	Color	7	Not Specified	No	No	Not Specified	[19]
	Color	17–20	Fattening Pigs	No	No	Not Specified	[20]
	Color	22	Fattening Pigs	No	No	Not Specified	[21]
	Color	22 or 23	Fattening Pigs	No	No	Not Specified	[22]
	Color	22	Fattening Pigs	No	No	Not Specified	[23]
	Color	29		No	No	3.7	[24]
	Color	3	Not Specified	No	No	15.0	[25]
	Color	10	Piglets	No	No	Not Specified	[26]
	Color	10	Piglets	No	No	Not Specified	[27]
	Color	10	Piglets	No	No	Not Specified	[28]
	Color	10	Piglets	No	No	Not Specified	[29]
	Color	10	Piglets	No	No	Not Specified	[30]
	Color	10	Piglets	No	No	Not Specified	[31]
	Color	12	Piglets	No	No	1–15	[32]
	Color	22	Piglets	No	No	Not Specified	[33]
	Infrared	1	Sow	No	No	8.5	[34]
	Infrared	~16	Fattening Pigs	No	No	Not Specified	[35]
	Infrared	6 or 12	Fattening Pigs	No	No	Not Specified	[36]
	Thermal	7	Piglets	No	No	Not Specified	[37]
3D	Stereo	1	Piglet	Not Specified	No	Not Specified	[38]
	Depth	1	29–139 kg Pig	Not Specified	No	Not Specified	[39]
	Depth	1	Sow	Yes	No	Not Specified	[40]
	Depth	1	Fattening Pig	Not Specified	No	Not Specified	[41]
	Depth	10	25 or 60 kg Pigs	Yes	No	Not Specified	[42]
	Depth	22	Piglets	Yes	No	15.1	[43]
	Depth	13	Piglets	Yes	Yes	494.7	Proposed Method

**Table 2:** Definition of key terms.

Category	Definition	Explanation
**Types of images**	$I_{input}$	Depth input image
	$I_{background}$	Background image
	$I_{interpolate}$	Image to which spatiotemporal interpolation is applied
	$I_{subtract}$	Image to which background subtraction is applied
	$I_{candidate}$	Image of candidates detected
	$I_{edge}$	Image of candidate edges
	$I_{outline}$	Image of outlines detected around standing-pigs
	$I_{overlap}$	Image overlapped between $I_{outline}$ and $I_{edge}$
	$I_{dilate}$	Image to which dilation operator is applied
	$I_{combine}$	Image combining $I_{overlap}$ with $I_{dilate}$
	$I_{output}$	Result image of standing-pigs
**Types of undefined values**	$UDF_{floor}$	Undefined values caused by slates on the floor
	$UDF_{outline}$	Undefined values for outlines generated around standing-pigs
	$UDF_{moving}$	Undefined values of moving noises in an input image
	$UDF_{limitation}$	Undefined values of Kinect’s limited distance and field-of-view

**Table 3:** Accuracy of standing-pig detection.

Method	Accuracy (%)
Proposed method	94.47
YOLO9000	86.25

**Table 4:** Results for the detection of standing-pigs during daytime and nighttime.

	No. of Undefined Pixels (%)	No. of Standing-Pigs	Proposed Method		YOLO9000 [50]	
			No. of True Standing-Pigs Detected	No. of False Standing-Pigs Detected	No. of Actual Standing-Pigs Detected	No. of False Standing-Pigs Detected
01:00	21.06	28	28	0	28	33
04:00	19.80	39	39	3	39	9
07:00	21.52	496	496	21	468	20
10:00	23.95	121	121	5	114	4
13:00	23.75	202	202	15	199	8
16:00	22.83	190	190	12	186	6
19:00	21.73	59	59	2	57	18
22:00	20.51	51	51	5	48	18
**Total**	**-**	**1186**	**1186**	**63**	**1139**	**116**

**Table 5:** Average processing speed for standing-pigs detection.

Method	Frames per Second
Proposed method	494.7
YOLO9000	87.0

## References

[1] Banhazi T., Lehr H., Black J., Crabtree H., Schofield P., Tscharke M., Berckmans D. (2012). Precision Livestock Farming: An International Review of Scientific and Commercial Aspects. Int. J. Agric. Biol..

[2] Neethirajan S. (2017). Recent Advances in Wearable Sensors for Animal Health Management. Sens. Bio-Sens. Res..

[3] Tullo E., Fontana I., Guarino M. Precision Livestock Farming: An Overview of Image and Sound Labelling. Proceedings of the 6th European Conference on Precision Livestock Farming (EC-PLF 2013).

[4] Matthews S., Miller A., Clapp J., Plötz T., Kyriazakis I. (2016). Early Detection of Health and Welfare Compromises through Automated Detection of Behavioural Changes in Pigs. Vet. J..

[5] Tscharke M., Banhazi T. (2016). A Brief Review of the Application of Machine Vision in Livestock Behaviour Analysis. J. Agric. Inform..

[6] Han S., Zhang J., Zhu M., Wu J., Kong F. Review of Automatic Detection of Pig Behaviours by using Image Analysis. Proceedings of the International Conference on AEECE.

[7] Wouters P., Geers R., Parduyns G., Goossens K., Truyen B., Goedseels V., Van der Stuyft E. (1990). Image-Analysis Parameters as Inputs for Automatic Environmental Temperature Control in Piglet Houses. Comput. Electron. Agric..

[8] Schofield C. (1990). Evaluation of Image Analysis as a Means of Estimating the Weight of Pigs. J. Agric. Eng. Res..

[9] Wongsriworaphon A., Arnonkijpanich B., Pathumnakul S. (2015). An Approach based on Digital Image Analysis to Estimate the Live Weights of Pigs in Farm Environments. Comput. Electron. Agric..

[10] Tu G., Karstoft H., Pedersen L., Jorgensen E. (2015). Illumination and Reflectance Estimation with its Application in Foreground. Sensors.

[11] Tu G., Karstoft H., Pedersen L., Jorgensen E. (2014). Segmentation of Sows in Farrowing Pens. IET Image Process..

[12] Tu G., Karstoft H., Pedersen L., Jorgensen E. (2013). Foreground Detection using Loopy Belief Propagation. Biosyst. Eng..

[13] Nilsson M., Herlin A., Ardo H., Guzhva O., Astrom K., Bergsten C. (2015). Development of Automatic Surveillance of Animal Behaviour and Welfare using Image Analysis and Machine Learned Segmentation Techniques. Animal.

[14] Brünger J., Traulsen I., Koch R. (2015). Randomized Global Optimization for Robust Pose Estimation of Multiple Targets in Image Sequences. Math. Model. Comput. Methods.

[15] Buayaui P., Kantanukul T., Leung C., Saikaew K. (2017). Boundary Detection of Pigs in Pens based on Adaptive Thresholding using an Integral Image and Adaptive Partitioning. CMU J. Nat. Sci..

[16] Lu M., Xiong Y., Li K., Liu L., Yan L., Ding Y., Lin X., Yang X., Shen M. (2016). An Automatic Splitting Method for the Adhesive Piglets’ Gray Scale Image based on the Ellipse Shape Feature. Comput. Electron. Agric..

[17] Ma C., Zhu W., Li H., Li X. Pig Target Extraction based on Adaptive Elliptic Block and Wavelet Edge Detection. Proceedings of the International Conference on Signal Processing Systems.

[18] Guo Y., Zhu W., Jiao P., Ma C., Yang J. (2015). Multi-object Extraction from Topview Group-Housed Pig Images based on Adaptive Partitioning and Multilevel Thresholding Segmentation. Biosyst. Eng..

[19] Guo Y., Zhu W., Jiao P., Chen J. (2014). Foreground Detection of Group-Housed Pigs based on the Combination of Mixture of Gaussians using Prediction Mechanism and Threshold Segmentation. Biosyst. Eng..

[20] Nasirahmadi A., Edwards S., Matheson S., Sturm B. (2017). Using Automated Image Analysis in Pig Behavioural Research: Assessment of the Influence of Enrichment Substrate Provision on Lying Behavior. Appl. Anim. Behav. Sci..

[21] Nasirahmadi A., Hensel O., Edwards S., Sturm B. (2017). A New Approach for Categorizing Pig Lying Behavior based on a Delaunay Triangulation Method. Animal.

[22] Nasirahmadi A., Hensel O., Edwards S., Sturm B. (2016). Automatic Detection of Mounting Behaviours among Pigs using Image Analysis. Comput. Electron. Agric..

[23] Nasirahmadi A., Richter U., Hensel O., Edwards S., Sturm B. (2015). Using Machine Vision for Investigation of Changes in Pig Group Lying Patterns. Comput. Electron. Agric..

[24] Ahrendt P., Gregersen T., Karstoft H. (2011). Development of a Real-Time Computer Vision System for Tracking Loose-Housed Pigs. Comput. Electron. Agric..

[25] Oczak M., Maschat K., Berckmans D., Vranken E., Baumgartner J. (2016). Automatic Estimation of Number of Piglets in a Pen during Farrowing, using Image Analysis. Biosyst. Eng..

[26] Ott S., Moons C., Kashiha M., Bahr C., Tuyttens F., Berckmans D., Niewold T. (2014). Automated Video Analysis of Pig Activity at Pen Level Highly Correlates to Human Observations of Behavioural Activities. Livest. Sci..

[27] Kashiha M., Bahr C., Ott S., Moons C., Niewold T., Tuyttens F., Berckmans D. (2014). Automatic Monitoring of Pig Locomotion using Image Analysis. Livest. Sci..

[28] Kashiha H., Bahr C., Ott S., Moons C., Niewold T., Odberg F., Berckmans D. (2014). Automatic Weight Estimation of Individual Pigs using Image Analysis. Comput. Electron. Agric..

[29] Kashiha M., Bahr C., Ott S., Moons C., Niewold T., Odberg F., Berckmans D. (2013). Automatic Identification of Marked Pigs in a Pen using Image Pattern Recognition. Comput. Electron. Agric..

[30] Kashiha M., Bahr C., Haredasht S., Ott S., Moons C., Niewold T., Odberg F., Berckmans D. (2013). The Automatic Monitoring of Pigs Water Use by Cameras. Comput. Electron. Agric..

[31] Viazzi S., Ismayilova G., Oczak M., Sonoda L., Fels M., Guarino M., Vranken E., Hartung J., Bahr C., Berckmans D. (2014). Image Feature Extraction for Classification of Aggressive Interactions among Pigs. Comput. Electron. Agric..

[32] Chung Y., Kim H., Lee H., Park D., Jeon T., Chang H. (2014). A Cost-Effective Pigsty Monitoring System Based on a Video Sensor. KSII Trans. Internet Inf..

[33] Zuo S., Jin L., Chung Y., Park D. An Index Algorithm for Tracking Pigs in Pigsty. Proceedings of the ICITMS.

[34] Khoramshahi E., Hietaoja J., Valros A., Yun J., Pastell M. (2014). Real-Time Recognition of Sows in Video: A Supervised Approach. Inf. Process. Agric..

[35] Costa A., Ismayilova G., Borgonovo F., Viazzi S., Berckmans D., Guarino M. (2014). Image-Processing Techniques to Measure Pig Activity in response to Climatic Variation in a Pig Barn. Anim. Prod. Sci..

[36] Brendle J., Hoy S. (2011). Investigation of Distances Covered by Fattening Pigs Measured with VideoMotionTracker. Appl. Anim. Behav. Sci..

[37] Cook N., Bench C., Liu T., Chabot B., Schaefer A. (2017). The Automated Analysis of Clustering Behavior of Piglets from Thermal Images in response to Immune Challenge by Vaccination. Animal.

[38] Shi C., Teng G., Li Z. (2016). An Approach of Pig Weight Estimation using Binocular Stereo System based on LabVIEW. Comput. Electron. Agric..

[39] Kongsro J. (2014). Estimation of Pig Weight using a Microsoft Kinect Prototype Imaging System. Comput. Electron. Agric..

[40] Lao F., Brown-Brandl T., Stinn J., Liu K., Teng G., Xin H. (2016). Automatic Recognition of Lactating Sow Behaviors through Depth Image Processing. Comput. Electron. Agric..

[41] Stavrakakis S., Li W., Guy J., Morgan G., Ushaw G., Johnson G., Edwards S. (2015). Validity of the Microsoft Kinect Sensor for Assessment of Normal Walking Patterns in Pigs. Comput. Electron. Agric..

[42] Zhu Q., Ren J., Barclay D., McCormack S., Thomson W. Automatic Animal Detection from Kinect Sensed Images for Livestock Monitoring and Assessment. Proceedings of the International Conference on Computational Cybernetics and Information Technology.

[43] Lee J., Jin L., Park D., Chung Y. (2016). Automatic Recognition of Aggressive Pig Behaviors using Kinect Depth Sensor. Sensors.

[44] Robert S., Dancosse J., Dallaire A. (1987). Some Observations on the Role of Environment and Genetics in Behaviour of Wild and Domestic Forms of Sus Scrofa (European Wild Boars and Domestic Pigs). Appl. Anim. Behav. Sci..

[45] Wood D., Vestergaard K., Petersen H. (1990). The Significance of Motivation and Environment in the Development of Exploration in Pigs. Biol. Behav..

[46] Ekkel E., Spoolder H., Hulsegge I., Hopster H. (2003). Lying Characteristics as Determinants for Space Requirements in Pigs. Appl. Anim. Behav. Sci..

[47] Mallick T., Das P.P., Majumdar A.K. (2014). Characterization of Noise in Kinect Depth Images: A Review. IEEE Sens. J..

[48] Open Source Computer Vision, OpenCV. http://opencv.org.

[49] Otsu N. (1979). Threshold Selection Method from Gray-Level Histograms. IEEE Trans. Syst. Man Cybern..

[50] Redmon J., Farhadi A. (2016). YOLO9000: Better, faster, stronger. arXiv.

[51] Greff K., Brandão A., Krauß S., Stricker D., Clua E. A Comparison between Background Subtraction Algorithms using a Consumer Depth Camera. Proceedings of the International Conference on Computer Vision Theory and Applications.

[52] Qiu X., Zhang S. Hand Detection for Grab-and-Go Groceries. In Stanford University Course Project Reports—CS231n Convolutional Neural Network for Visual Recognition. http://cs231n.stanford.edu/reports.html.

[53] Bottger T., Follmann P., Fauser M. (2017). Measuring the Accuracy of Object Detectors and Trackers. arXiv.

[54] Lin B.S., Su M.J., Cheng P.H., Tseng P.J., Chen S.J. (2015). Temporal and Spatial Denoising of Depth Maps. Sensors.

[55] He Y., Liang B., Zou Y., He J., Yang J. (2017). Depth Errors Analysis and Correction for Time-of-Flight (ToF) Cameras. Sensors.

